# Intrinsic stability and oligomerization dynamics of DNA processivity clamps

**DOI:** 10.1093/nar/gku255

**Published:** 2014-04-09

**Authors:** Jennifer K. Binder, Lauren G. Douma, Suman Ranjit, David M. Kanno, Manas Chakraborty, Linda B. Bloom, Marcia Levitus

**Affiliations:** 1 Department of Chemistry and Biochemistry and Biodesign Institute, Arizona State University, Tempe, AZ 85287-5601, USA; 2 Department of Biochemistry and Molecular Biology, University of Florida, Gainesville, FL 32610-0245, USA

## Abstract

Sliding clamps are ring-shaped oligomeric proteins that are essential for processive deoxyribonucleic acid replication. Although crystallographic structures of several clamps have been determined, much less is known about clamp structure and dynamics in solution. Here, we characterized the intrinsic solution stability and oligomerization dynamics of the homodimeric *Escherichia coli* β and the homotrimeric *Saccharomyces cerevisiae* proliferating cell nuclear antigen (PCNA) clamps using single-molecule approaches. We show that *E. coli* β is stable in solution as a closed ring at concentrations three orders of magnitude lower than PCNA. The trimeric structure of PCNA results in slow subunit association rates and is largely responsible for the lower solution stability. Despite this large difference, the intrinsic lifetimes of the rings differ by only one order of magnitude. Our results show that the longer lifetime of the *E. coli* β dimer is due to more prominent electrostatic interactions that stabilize the subunit interfaces.

## INTRODUCTION

Replication of genomes requires the coordinated activities of many enzymes and proteins. At the heart of replisomes are deoxyribonucleic acid (DNA) polymerases that catalyze the synthesis of daughter strands, but these enzymes are unable to rapidly synthesize long stretches of DNA due to frequent dissociation from the strand being copied (1–3). In all domains of life, the lifetime of a polymerase on DNA is substantially increased by binding to a sliding clamp (4). By virtue of their ability to diffuse along DNA, ring-shaped sliding clamps act as mobile attachment points to anchor polymerases to the template as they synthesize DNA (5,6). The ability of a clamp to confer high processivity to a DNA polymerase depends on the lifetime of the closed ring structure. Transient opening or dissociation of clamp subunits would break the ring, allowing the polymerase–clamp complex to dissociate from DNA. Most clamps are inherently stable rings that form long-lived complexes with DNA. The lifetimes on DNA of the *Escherichia coli* β clamp and human proliferating cell nuclear antigen (PCNA) are about 100–170 and 35 min at 37°C, respectively (7,8). An exception to this rule is the bacteriophage T4 gp45 sliding clamp that adopts an open ring structure in solution and does not stably bind DNA in the absence of the phage polymerase (9,10). The secondary and tertiary structures of sliding clamps are remarkably conserved from bacteria to humans, even though these proteins have little homology at the primary sequence level (11–15). Sliding clamps contain six globular domains of similar fold that, in the case of the bacterial clamp, are linked in two units of three to form a dimer. In contrast, the eukaryotic, archaeal and viral clamps are trimers, with each monomer containing two globular domains. Whether or not this difference in the stoichiometry of subunits has any consequences in terms of clamp stability and dynamics is an open question.

Although a great deal is known about the static structure of sliding clamps, much less is known about clamp dynamics in solution (10,16,17). Subunit dynamics has important implications in the loading and unloading pathways of clamps, and is therefore a key aspect of their physiological function. Here, we describe single-molecule studies designed to characterize the solution oligomerization dynamics and intrinsic stability of the homodimeric *E. coli* β and the homotrimeric *Saccharomyces cerevisiae* PCNA sliding clamps. A detailed kinetic analysis of the oligomerization dynamics of both clamps reveals that subunit stoichiometry is the major contributing factor for the three orders of magnitude lower equilibrium dissociation constant of β and the significantly faster equilibration rates of PCNA. Despite these differences, we established that the dissociation lifetime of β, which is a direct measure of intrinsic stability of the ring, is only about one order of magnitude higher than PCNA. This difference is not a consequence of the different subunit stoichiometry, but rather reflects the more prominent role of electrostatic interactions between the subunits of β than PCNA.

## MATERIALS AND METHODS

### Purification of β and PCNA

To create samples for fluorescence correlation spectroscopy (FCS) measurements, surface Cys residues were replaced with Ser (Cys-260 and Cys-333 in β; Cys-22, Cys-62 and Cys-81 in PCNA), and a single Cys residue was engineered at a specific site, Ile-305 to Cys in β and Ile-181 to Cys in PCNA, to allow for site-specific labeling with a maleimide derivative of TMR. For subunit exchange and single-molecule experiments, two residues were engineered into a second β mutant, Arg-103 to Cys and Ile-305 to Cys, to allow for doubly labeling this mutant. All clamps were over-expressed in *E. coli* and purified as described previously for β (18,19) and PCNA (20). Site-directed mutagenesis and fluorescent labeling of β-TMR_1_ (C260S/C333S/I305C), β-TMR_2_ (C260S/C333S/R103C/I305C) and PCNA-TMR_1_ (C22S/C62S/C81S/I181C) were done as described previously (20,21).

### Fluorescent labeling of β and PCNA

Both β and PCNA were labeled with TMR (Molecular Probes, Invitrogen). Sliding clamps (2 mg) were incubated in the dark with 20 equivalents of fluorophore per cysteine residue for 2 h at room temperature followed by 4°C overnight in a buffered solution containing 1 mM TCEP and 8% dimethyl sulfoxide. Buffer solutions contained 20 mM Tris–HCl (pH 7.5) and 0.5 mM ethylenediaminetetraacetic acid (EDTA) for β or 30 mM 4-(2-Hydroxyethyl)-1-piperazineethanesulfonic acid (HEPES, pH 7.5), 0.5 mM EDTA and 150 mM NaCl for PCNA. Labeled protein was separated from excess fluorophore by gel filtration chromatography using Bio-Gel P-6DG (Bio-Rad) desalting resin. Proteins were further purified by anion exchange chromatography on a 1-ml HiTrap Q-Sepharose column (GE Healthcare). β was eluted with a linear gradient of 0.1–1 M NaCl in buffer containing 20 mM Tris–HCl (pH 7.5) and 0.5 mM EDTA. PCNA was eluted with a linear gradient of 0.15–1 M NaCl in buffer containing 30 mM HEPES (pH 7.5) and 0.5 mM EDTA. Proteins were dialyzed overnight against 20 mM Tris–HCl (pH 7.5), 0.5 mM EDTA and 10% glycerol for β and 30 mM HEPES (pH 7.5), 0.5 mM EDTA, 150 mM NaCl, 2 mM DTT (dithiothreitol), and 10% glycerol for PCNA. Proteins were aliquoted and stored at −80°C. The protein concentrations were determined by a Bradford-type Assay (Bio-Rad) using native β or PCNA standards quantified by denatured *A*_280_. The concentration of TMR was calculated from the absorbance measured at 555 nm using an extinction coefficient of 98 000 M^−1^ cm^−1^. Both PCNA and β were labeled at one site per subunit at positions I181C and I305C, respectively. Typical labeling yields were 70% for both β and PCNA.

### FCS and single-molecule measurements

We measured single-molecule fluorescence traces and FCS decays using a home-built optical setup. A 532 nm CW laser (Coherent Compass 215M-10, Santa Clara, CA, USA) was attenuated to 100 μW and focused on the sample using a 1.4 NA objective lens (Olympus PlanApo 100X/1.4 NA oil). The fluorescence from the sample was collected by the same objective and then passed through a 50 μm pinhole, creating a confocal volume size of ∼5 fl. The fluorescence was then detected using an avalanche photodiode (Perkin-Elmer Optoelectronics, SPCM-AQR14). Autocorrelation decays (*G*(*τ*)) were measured with a multiple-tau digital correlator (ALV-5000/60X0, ALV, Germany). A PCI-6602 acquisition card (National Instruments, Austin, TX, USA) was used to acquire the single-molecule data. Solutions were prepared in a buffer containing 20 mM Tris–HCl pH 7.5, 50 mM NaCl and 0.1 mg/ml BSA (bovine serum albumin). Variable concentrations of NaCl were used in some experiments as indicated in the text. For FCS, we used 1 nM solutions of singly labeled clamps (i.e. one label per subunit). Equilibrium experiments were performed by mixing with variable concentrations of unlabeled protein to achieve total concentrations in the 1 nM–1 μM range. Solutions were incubated for 24 h at room temperature prior to the FCS measurements. For the kinetic experiments, we prepared a 1 nM solution of labeled protein by diluting from a concentrated stock (>10 μM) and incubated the solution at room temperature in an Eppendorf tube. About 30 μl of this solution were taken at regular intervals to acquire an FCS decay. For single-molecule fluorescence, we incubated a 10 pM solution of doubly labeled β (β-TMR_2_) in an Eppendorf tube for 8 h. We took about 30 μl of solution at regular intervals and acquired single molecule traces with 1 ms resolution for 10 min. Frequency histograms acquired at increasing incubation times were constructed from each 10 min trace (6 × 10^5^ data points). Because the alignment of the optical setup cannot be kept invariable over weeks of operation, the histograms may be biased toward higher or lower values based on the alignment of the instrument. For these reason, to compare experiments performed on different days we normalized the values of *f* (*k* > 6) by the average count rate obtained during that particular experiment (typically ∼1.5–2 counts/ms).

### Analysis of FCS data

The autocorrelation function measured in FCS experiments with a monodisperse sample diffusing freely in solution is:

(1) \begin{equation*} G(\tau ) = \frac{1}{{\left\langle N \right\rangle }}\frac{1}{{1 + \tau /\tau _{\rm D}}} \end{equation*}

where *τ* is the correlation lag time, *τ*_D_ is the characteristic diffusion time and 〈*N*〉^−1^ is the mean number of fluorescent particles in the observation volume. We note that Equation (1) assumes that the axial dimension of the setup (*z*) is much larger than its radial dimension (*r*), which is the case in our instrument. The characteristic diffusion time is related to the particle's diffusion coefficient (*D*) by *τ*_D_ = 4*r^2^*/*D*. The values of *τ*_D_ were obtained from non-linear least squares fitting of the experimental autocorrelation decays.

In the case of a polydisperse sample, the total autocorrelation function can be expressed as the sum of the autocorrelation functions of the individual components weighted by the square of the brightness of each particle:

(2) \begin{equation*} G(\tau ) = \frac{{\sum\limits_{i = 1}^n {B_i^2 N_i } \left( {1 + \tau /\tau _{\rm D}^i } \right)^{ - 1} }}{{\left( {\sum\limits_{i = 1}^n {B_i N_i } } \right)^2 }} \end{equation*}

where *n* represents the number of species present in solution, *N_i_* represents the number of particles of species *i* present in the observation volume on average, *B_i_* represents its brightness and *τ^i^*_D_ represents its characteristic diffusion time.

The total autocorrelation decay of a mixture of trimer, dimer and monomer (described by Equation (2)) is experimentally indistinguishable from the autocorrelation decay of a monodisperse sample (Equation (1)). Therefore, the experimental data was fitted with Equation (1), which contains the least amount of fitting parameters (see Supplementary Figure S1 for example). Fitting with Equation (2) to attempt to extract the values of *N_i_* (*i* = monomer, dimer and trimer) directly results in non-physical outcomes due to overfitting (excessive number of fitting parameters). The *τ*_D_ values recovered from the fits using Equation (1) are apparent diffusion times (*τ*_APP_) that depend on the diffusion times of the individual oligomeric species and their relative concentrations. The values reported in Figure 2B represent averages of 3–5 independent determinations for each concentration, and error bars represent standard deviations.

**Figure 1.**
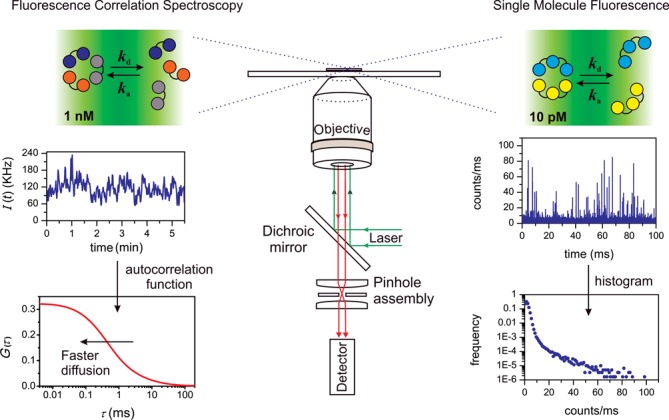
Experimental setup. We investigated the oligomerization equilibrium and dynamics of PCNA (a trimer) and β (a dimer) using FCS and single molecule fluorescence measurements. Experiments were performed in a confocal single-molecule setup. FCS decays were acquired with 1 nM labeled and variable concentrations of unlabeled protein. Fluctuations in fluorescence intensity (middle left) were analyzed in terms of the autocorrelation function (bottom left), which shifts toward faster times as the oligomeric proteins dissociate into faster-diffusing monomers. Single-molecule traces (middle right) were measured with 10 pM labeled protein solutions. Bursts of fluorescence are generated when individual fluorescent molecules traverse the observation volume. Single-molecule data was analyzed in terms of histograms representing the frequency of bursts containing *k* photons per millisecond (bottom right).

**Figure 2.**
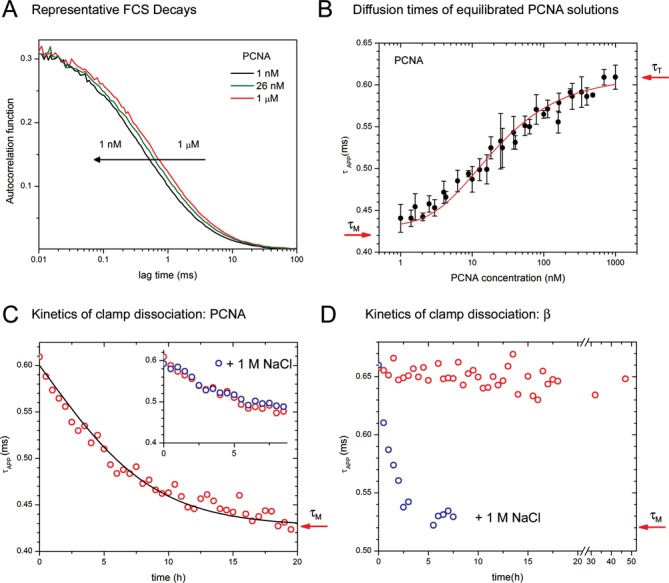
FCS experiments to determine equilibrium and kinetic parameters of the dissociation equilibrium of PCNA and β. All experiments were performed in a buffer containing 50 mM NaCl unless indicated. (**A**) Representative FCS decays obtained with PCNA at the total concentration indicated in the figure. All traces were measured with equilibrated solutions prepared by mixing 1 nM labeled PCNA with the amount of unlabeled PCNA needed to achieve the desired total concentration. The decays were overlapped at short lag times to stress the differences in diffusion time. Actual amplitudes were all in the 0.28–0.4 range. (**B**) *τ*_APP_ values obtained by fitting the FCS decays of PCNA solutions equilibrated for 24 h. Concentrations are expressed in terms of PCNA trimers, and arrows indicate the *τ*_D_ values of the pure trimer and monomer. Error bars represent the standard deviation of at least three independent measurements. The best fit to the data (red line) was obtained with *K*_d_ = (2100 ± 500) nM^2^. (**C**) *τ*_APP_ values obtained by fitting the FCS decays of a freshly diluted 1 nM PCNA solution as a function of incubation time (red circles). The arrow indicates the diffusion time of the monomer. The best fit to the data (black line) was obtained with *k*_d_ = (6.1 ± 0.5) × 10^−5^ s^−1^. Inset: comparison between experiments performed in 50 mM (red) and 1 M NaCl (blue). (**D**) *τ*_APP_ values obtained from the FCS decays of a freshly diluted 1 nM solution of β containing 50 mM NaCl (red) or 1 M NaCl (blue) as a function of incubation time (red circles). The arrow indicates the diffusion time of the monomer.

The equilibration of a mixture of unlabeled and labeled protein results in a distribution of labeled, unlabeled and partially labeled trimers and dimers, which dissociate into a mixture of labeled and unlabeled monomers. The brightness of each of these species (*B_i_*, Equation (2)) is determined by the number of fluorescently labeled monomers present in each particle, and can be expressed in terms of the concentrations of labeled and unlabeled protein as described in detail in previous work (22) to give:

(3) \begin{equation*} G(\tau ) = \frac{{\begin{array}{&ast;{20}c} {\alpha _1 \left( {1 + \tau /\tau _{\rm M} } \right)^{ - 1} + \alpha _2 \left( {1 + \frac{{fC_{\rm L}}}{C}} \right)\left( {1 + \tau /\tau _{\rm D}} \right)^{ - 1} } \\ { + \alpha _3 \left( {1 + 2\frac{{fC_{\rm L} }}{C}} \right)\left( {1 + \tau /\tau _{\rm T}} \right)^{ - 1} } \\ \end{array}}}{{3V_{{\rm eff}} fC_{\rm L} N_{{\rm AV}} }} \end{equation*}

where *α*_1_, *α*_2_ and *α*_3_ represent the mole fractions of monomer, dimer and trimer $\left( {\alpha _1 = \frac{{[{\rm M}]}}{{3C}},\alpha _2 = \frac{{2[{\rm D}]}}{{3C}},\alpha _3 = \frac{{[{\rm T}]}}{C}} \right)$, *f* is the efficiency of the labeling reaction, *C*_L_ is the concentration of labeled protein (fixed at 1 nM in all experiments), *C* is the total protein concentration (unlabeled + labeled) expressed as trimers, *N*_AV_ is Avogadro's number and *V*_eff_ is the optical effective volume. We note that although *V*_eff_ can be calibrated with a standard, its value affects only the amplitude of the autocorrelation function and not the parameter of interest (*τ*_APP_).

To obtain the value of the dissociation constant of the trimer–monomer equilibrium from the experimental data shown in Figure 2B, we created an algorithm that iterates the value of *K*_d_ until the square of the residuals between the experimental and calculated *τ*_APP_ values is minimized. In the first step, an initial *K*_d_ value is assumed and the values of [M] and [T] are calculated for each of the 50 protein concentrations by solving the following algebraic equations:

\begin{equation*} K_{\rm d} = \frac{{[{\rm M}]^3 }}{{[{\rm T}]}} \end{equation*}

\begin{equation*} C = [{\rm T}] + [{\rm M}]/3 \end{equation*}

In the second step, the values of [M] and [T] are used in Equation (3) to calculate the theoretical autocorrelation decays that are expected at each protein concentration. These theoretical *G*(*τ*) decays are then fitted with Equation (1) to obtain the *τ*_APP_ values that are expected for the assumed *K*_d_ value. The *K*_d_ is then modified and the algorithm repeated until the calculated *τ*_APP_ values provide the best fit to the experimental points.

The same overall methodology was used to evaluate the existence of possible dimeric intermediates in the assembly mechanism of PCNA. In this case, we calculated [M], [D] and [T] for each of the 50 total protein concentrations from

\begin{equation*} K_{{\rm d}1} = \frac{{[{\rm M}][{\rm D}]}}{{[{\rm T}]}},\quad K_{{\rm d}2} = \frac{{[{\rm M}]^2 }}{{[{\rm D}]}} \end{equation*}

\begin{equation*} C = [{\rm T}] + [{\rm D}]/2 + [{\rm M}]/3 \end{equation*}

and used Equation (3) to calculate the theoretical autocorrelation decays that are expected at each protein concentration. These theoretical *G*(*τ*) decays are then fitted with Equation (1) to obtain the *τ*_APP_ values that are expected for the assumed *K*_d1_ and *K*_d2_ values. The *K*_d_ values are then modified until the calculated *τ*_APP_ values provide the best fit to the experimental points.

To obtain the dissociation and association kinetic rate constants (*k*_d_ and *k*_a_) from the data of Figure 2C, we first assume a value of *k*_d_, and we iterate until the calculated time-dependent *τ*_APP_ values match the experimental data. In the first step, the values of [T] and [M] are calculated for each of the ca. 40 time points for the assumed value of *k*_d_ by solving the following set of algebraic and differential equations:

\begin{equation*} \begin{array}{&ast;{20}c} {\frac{{d[{\rm T}]}}{{dt}} = - k{}_{\rm d}[{\rm T}] + \frac{{k{}_{\rm d}}}{{K{}_{\rm d}}}[{\rm M}]^3 } \\ {C = [{\rm T}] + [{\rm M}]/3} \\ \end{array} \end{equation*}

Here, *K*_d_ is the dissociation constant we determined in the previous analysis, *C* = 1 nM in all experiments, and we set the association rate constant to *k*_a_ *= k*_d_/*K*_d_. The differential equation was solved numerically for each time point to obtain the time-dependent concentrations of monomer and trimer consistent with the assumed dissociation rate constant. As in the analysis of the equilibrium data, in the second step the algorithm generates the total autocorrelation function expected at each time point (Equation (3)), and fits the resulting curve with Equation (1) to calculate the *τ*_APP_ values. A plot of *τ*_APP_ versus time is then generated and compared with the experimental data. The procedure is repeated by varying *k*_d_ until the calculated data provides the best fit to the experimental points.

### Fluorescence lifetime measurements

**Figure 3.**
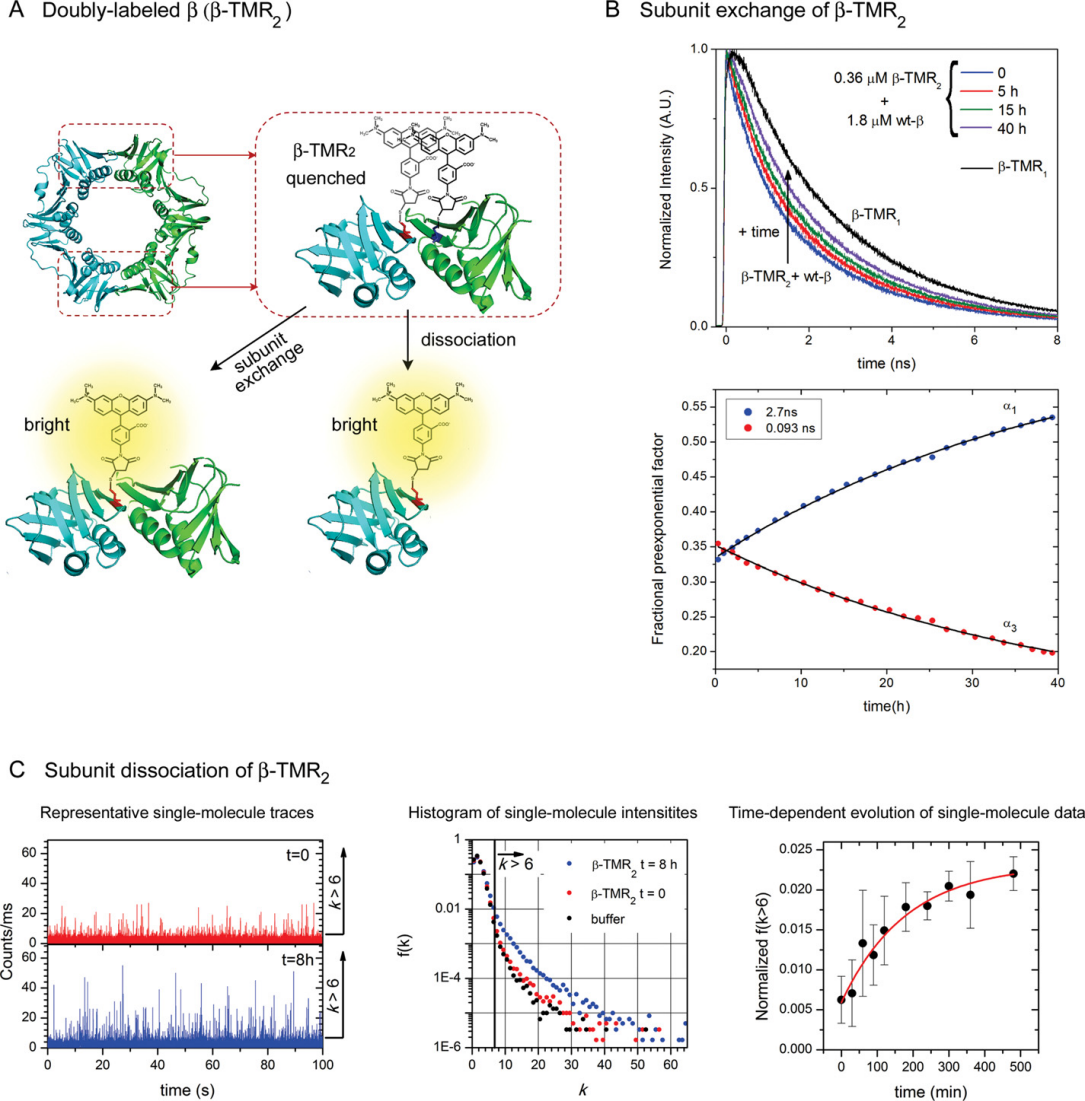
(**A**) Labeling strategy used to generate β-TMR_2_ samples for single-molecule and subunit exchange experiments. Ribbon diagram of the *Escherichia coli* β dimer (PDB ID: 1MMI). Residues R103 and I305 on each subunit (highlighted in blue and red, respectively) were mutated to Cys and labeled with TMR to create the doubly labeled samples (β-TMR_2_). Note that the protein contains a pair of TMR molecules at each of its interfaces (top and bottom). TMR molecules are drawn to scale. β-TMR_2_ particles are not fluorescent due to TMR self-quenching. Fluorescence is recovered after addition of excess wt-β, which results in clamps containing only one labeled monomer (exchange experiments), or as the protein dissociates into monomers at low concentrations (single-molecule experiments). (**B**) Time-resolved fluorescence experiments to determine the kinetics of subunit exchange of the β-clamp. Representative normalized fluorescence intensity decays obtained with a mixture of 0.36 μM β-TMR_2_ and 1.8 μM wt-β (top plot). Fluorescence decays were acquired for 40 h at regular intervals after mixing (*t* = 0). The decay of single-labeled PCNA (β-TMR_1_) is shown for comparison. All decays were fitted with three exponential terms as described in ‘Materials and Methods’ section. The bottom plot shows the fractional amplitudes (*α*) obtained from fitting all fluorescence intensity decays globally with three exponential terms (*τ*_1_ = 2.7 ns, *τ*_2_ = 1.1 ns and *τ*_3_ = 0.093 ns, see ‘Materials and Methods’ section). The amplitude that corresponds to *τ*_2_ remains fairly constant during the experiment and it is not shown for clarity. A relaxation time of 43 h was obtained by fitting the data with an exponential model (solid lines). (**C**) Dissociation of β at the single-molecule level. Representative single-molecule fluorescence traces measured with 10 pM β-TMR_2_ right after diluting from a concentrated stock (*t* = 0) and after 8 h of incubation (left). Bursts with >6 photons/ms (*k* > 6) are due to a fluorescently labeled protein particle with a probability higher than 99.5%. The middle panel shows frequency histograms of the data obtained with pure buffer (black) and β-TMR_2_ at *t* = 0 (red) and *t* = 8 h (blue). The increase in the frequency of bursts with large numbers of photons indicates dissociation of the protein dimer (quenched fluorescence) into monomers (fluorescent). The right panel shows the time evolution of the fraction of bursts with *k* > 6 normalized by the average fluorescence intensity (see ‘Materials and Methods’ section). A 3 h lifetime was obtained from an exponential fit to the data (red line).

We acquired fluorescence intensity decays at room temperature using the time-correlated single photon counting technique. We fully described the instrument elsewhere (23). Briefly, we used 540 nm excitation (FWHM ∼ 50 ps) and 590 nm emission (collected at the magic angle). Each decay was collected for 15 min. Data was analyzed with a software written in-house (ASUFIT, URL: www.public.asu.edu/~laserweb/asufit/asufit.html). All traces were deconvoluted from the instrumental response function and fitted globally with the sum of three exponentials:

\begin{equation*} I(t,T) = A_1 (T)e^{ - t/\tau _1 } + A_2 (T)e^{ - t/\tau _2 } + A_3 (T)e^{ - t/\tau _3 } \end{equation*}

where *T* indicates the incubation time (hour timescale) and *t* is the fluorescence decay time (nanosecond timescale). The three lifetimes *τ*_1–3_ were shared among all data sets (about 30) and the amplitudes *A*_1–3_ were treated as independent. The fractional amplitudes shown in Figure 3B were calculated as *α_i_* = *A_i_*/(*A*_1_ + *A*_2_ + *A*_3_). Examples of fitted data are shown in Supplementary Figure S4.

## RESULTS

### FCS assay for clamp oligomerization dynamics

To investigate the equilibrium and kinetics of clamp oligomerization, we developed a single-molecule approach to monitor changes in subunit composition in real time. The assay is based on the technique known as FCS, which consists in measuring and analyzing fluctuations in fluorescent intensity of a small population of fluorescently labeled protein particles (24,25). Molecules close to the focus are excited and detected with an efficiency greater than those that are farther away, and therefore, Brownian motion results in fluorescence intensity fluctuations on the timescale of molecular diffusion (Figure 1). As is customary in the field, we define the characteristic diffusion time as *τ*_D_ = 4*r^2^*/*D*, where *D* is the diffusion coefficient of the fluorescently labeled protein particle and *r* is the radial dimension of the observation volume created by the laser (26). According to Stokes–Einstein's equation, *τ*_D_ is proportional to the hydrodynamic radius of the diffusing particle and consequently is proportional to the cube root of the molecular weight of the protein (*τ*_D_ ∝ *M*_w_^1/3^). The value of *τ*_D_ can be obtained from non-linear fitting of the experimental autocorrelation decay, *G*(*τ*), which is a function that provides a quantitative measure of the temporal behavior of the measured fluctuations in fluorescence intensity (see ‘Materials and Methods’ section and Figure 1). Therefore, the autocorrelation function is expected to shift toward faster timescales (smaller *τ*_D_) as the protein dissociates into monomers (Figures 1 and 2A).

To create samples for FCS measurements, we labeled a single Cys residue engineered in each subunit of the *E. coli* β and the *S. cerevisiae* PCNA clamps with a TMR fluorescent probe (see ‘Materials and Methods’ section). The mutations do not affect the activity of the clamps or interactions with the clamp loader (20,21). FCS decays for fluorescently labeled clamps were acquired and analyzed in order to measure the characteristic diffusion times of the protein solutions. These values were further analyzed to calculate the concentrations of the different oligomeric species needed to calculate equilibrium and kinetic constants.

### Oligomerization equilibrium of PCNA

FCS measurements are restricted to concentrations below ca. 100 nM because the amplitude of the autocorrelation decay is inversely proportional to the concentration of the fluorescently labeled sample (Equation (1) and Supplementary Figure S1). Therefore, to be able to determine *τ*_D_ values at higher protein concentrations, we kept the concentration of labeled PCNA constant at 1 nM and adjusted the total concentration of protein to the desired value (1 nM–1 μM) by mixing with unlabeled PCNA. All solutions were incubated for 24 h at room temperature prior to the FCS measurements to equilibrate the labeled and unlabeled proteins, and to ensure complete equilibration between the oligomeric species that exist at the different protein concentrations (monomers, trimers and potentially dimers). Consistent with trimer dissociation, the FCS decays of the ‘equilibrated’ samples shift to shorter timescales as the concentration of PCNA decreases (Figure 2A).

All FCS decays (∼50 concentrations in the 1 nM–1 μM range) were fitted with Equation (1). The *τ*_D_ value recovered from the fit will be referred to as the ‘apparent *τ*_D_’ (*τ*_APP_) to stress the fact that, in principle, it does not represent the diffusion time of any one species present in the solution. Instead, the apparent diffusion time is determined by the diffusion times of the monomer and trimer (*τ*_M_ and *τ*_T_, respectively) and their concentrations ([M] and [T]). The possible existence of a dimeric intermediate will be omitted in the analysis below, and will be discussed in detail later. As shown in Figure 2B, the value of *τ*_APP_ decreases from 0.61 ms (1 μM PCNA) to ∼0.44 ms (1 nM PCNA) as the trimeric protein dissociates into smaller subunits. The ratio of these *τ*_APP_ values is 1.39, slightly less than the theoretical trimer/monomer ratio of *τ*_T_/*τ*_M_ = 3^1/3^ ≈ 1.44. The *τ*_APP_ plot has an approximately sigmoidal shape with an inflection point at 11.8 nM. Although this number provides a reasonable approximation of the clamp concentration at which the monomer and trimer co-exist in equilibrium at similar concentrations, it should be stressed that there is no theoretical basis for fitting this data with a sigmoid function or for assigning the inflection point as the *K*_d_ of the reaction. Instead, a rigorous evaluation of Figure 2B should focus on the relationship between the observable *τ*_APP_ and the quantities required to calculate *K*_d_: [M] and [T].

To obtain the value of the dissociation constant from the experimental data shown in Figure 2B, we created an algorithm based on a methodology we developed in previous work (22). The procedure, described in detail in ‘Materials and Methods’ section, is based on the fact that the experimental FCS decays fit well with a single *τ*_D_ value even when two or more species of different molecular masses (e.g. the trimer and the monomer) contribute to the measured signal. At concentrations of PCNA high enough for dissociation to be negligible, only the trimer exists in solution, and the measured *τ*_APP_ equals the characteristic diffusion time of the trimer (*τ*_T_). At lower concentrations, however, both the trimer and the monomer contribute to *τ*_APP_, and the analysis entails finding the amount of trimer and monomer in the solution that result in a given measured *τ*_APP_ value. A global analysis of the experimental concentration-dependent *τ*_APP_ values of Figure 2B gives *K*_d_ = 2100 nM^2^. The theoretical curve that corresponds to this *K*_d_ value is shown as a solid line superimposed on the experimental data. An error of ±500 nM^2^ was estimated by overlapping the experimental data with curves generated with different *K*_d_ values in the vicinity of 2100 nM^2^. In order to appreciate the physical meaning of a *K*_d_ with units of concentration squared, we recall that *K*_d_ = [M]^3^/[T], and therefore, the concentration of monomer at which [M] = [T] equals *K*_d_^1/2^ = 46 nM. This quantity has no thermodynamic meaning, but it is useful for comparing the dissociation constants of a trimer and a dimer directly.

The dissociation constant of the sliding clamp from bacteriophage T4 (gp45, a trimer) was determined from equilibrium ultracentrifugation experiments as 0.21 μM^2^ (9), indicating that PCNA is significantly more stable as a trimer than gp45. This is consistent with previous reports (7) and with the fact that gp45 was found to be partially open in solution (9,10), but no evidence of spontaneous opening of PCNA exists. Our data can be adequately analyzed in terms of a mechanism that involves only the monomer and the trimer, implying that if a dimeric intermediate exists, it is present at low concentrations and cannot be resolved from the experimental FCS data. A cooperative monomer-to-trimer assembly was also observed for gp45 (9). From the mechanistic point of view, however, the simultaneous encounter of three monomers is statistically implausible and a transient dimer is expected to be an intermediate in the assembly of the trimer.

\begin{equation*} \begin{array}{&ast;{20}c} {{\rm T}\mathop \rightleftharpoons\limits^{K_{{\rm d}1} } {\rm D} + {\rm M}} \\ {{\rm D}\mathop \rightleftharpoons\limits^{K_{{\rm d}2} } 2{\rm M}} \\ \end{array} \end{equation*}

To evaluate the highest concentration of dimer that is consistent with the experimental results, we analyzed the experimental data of Figure 2B with an algorithm that incorporates a dimer as an intermediate in the dissociation mechanism of PCNA. Our mathematical analysis shows that values of *K*_d2_ > ca. 10*K*_d1_ are required to describe the experimental dissociation curve. As shown in Supplementary Figure S2, lower *K*_d2_ values are inconsistent with the complete dissociation from trimer to monomer, we observe in three decades of concentration. Values of *K*_d1_ = 14.5 nM and *K*_d2_ = 145 nM give the highest concentration of dimer that is compatible with our experimental data. Under these conditions, the fraction of monomers engaged in dimeric particles (*α*_2_ = 2[D]/3*C*) is highest at a protein concentration *C* = 22 nM, and equals *α*_2_ = 0.15. This fraction represents an upper bound, and lower concentrations of dimer are expected when *K*_d2_ > 10*K*_d1_. Although the actual concentration of dimer in equilibrium depends on the values of *K*_d2_ and *K*_d1_, which we cannot determine with precision beyond the limit *K*_d2_ > 10*K*_d1_, the results of the simulations show that the concentration of dimer is maximal ∼10–30 nM total PCNA concentration independently of the actual values of the dissociation constants. In other words, if the dimer exists in equilibrium with the trimer and monomer, its concentration is highest ∼20 nM PCNA, and it never represents >15% of the total protein concentration.

### Oligomerization kinetics of PCNA

To determine the kinetic rate constants of the monomer–trimer equilibrium process, we acquired FCS decays of a ‘freshly diluted’ 1 nM labeled PCNA solution at regular time intervals. This concentration was chosen because it falls in the range of optimal signal-to-noise ratio in FCS experiments, and because we expect almost complete dissociation in equilibrium conditions (Figure 2B). We diluted the fluorescently labeled protein from a concentrated stock (ca.15 μM) at time *t* = 0, and recorded FCS decays over several hours (see Supplementary Figure S1 for example). As shown in Figure 2C, *τ*_APP_ decreases from an initial value of 0.61 ms, consistent with the trimer, to values ∼0.42 ms. The *τ*_APP_ measured at 20 h is very close to the value expected for the monomer (indicated with an arrow). The addition of 1 M NaCl to the buffer did not result in noticeable changes in the kinetics of dissociation (Figure 2C, inset). To obtain the kinetic rates of dissociation and association (*k*_d_ and *k*_a_) from the time-dependent changes in *τ*_APP_, we developed an algorithm based on the same principles we described above to analyze the equilibrium data. The procedure, described in detail in ‘Materials and Methods’ section, starts by assuming a value for the dissociation rate constant (*k*_d_), and then modifies this value in each iteration until the calculated time-dependent *τ*_APP_ values match the experimental data. The best fit to the experimental points was obtained for *k*_d_ = (6.1 ± 0.5) × 10^−5^ s^−1^. The reciprocal of the dissociation rate constant represents the lifetime of the trimeric particle, and equals *τ*_T_ = 4.5 ± 0.4 h. The association rate constant was determined as *k*_a_ *= k*_d_/*K*_d_ = (3 ± 1) × 10^10^ s^−1^ M^−2^. This is an apparent association rate constant of trimer formation because chemical association reactions occur as bimolecular encounters. The results described in the previous section show that the formation of the trimer is highly cooperative, and that the stability of the dimeric intermediate is low. This indicates that the lifetime of the dimer is short, and that once formed, it will dissociate unless a third monomer exists in close proximity. The rate of formation of the trimer is, therefore, limited by the rate of encounters between a short-lived dimer and another monomer.

The same kinetic experiments described above for PCNA were carried out with 1 nM singly labeled β (Figure 2D). Because β is a dimer, complete dissociation is expected to result in a decrease of *τ*_APP_ by a factor of 2^1/3^ (indicated by a red arrow). No changes in *τ*_APP_ were measured even after 48 h of incubation or in buffers containing up to 150 mM NaCl (Supplementary Figure S3). Mathematical modeling shows that these results are consistent with values of *K*_d_ < 0.3 nM, as higher values would result in a measurable decrease in *τ*_APP_. This is consistent with a previous estimate of *K*_d_ < 50 nM reported by Jeruzalmi *et al.* (27). In sharp contrast to PCNA, β dimer dissociation depends on ionic strength and is complete after 5 h incubation in experiments with 1 M NaCl (Figure 2D). Ionic strength was also recently shown to promote subunit dissociation in micromolar solutions of β (28). These experiments, therefore, indicate that β is stable as a dimer at nanomolar concentrations in buffers containing biochemically relevant concentrations of NaCl. Performing FCS experiments at lower concentrations, however, is difficult because the average number of fluorescently labeled particles in the optical observation volume drops well below one and only background signal is observed most of the time. For these reasons, we investigated the equilibrium and dynamics of dimerization of β with the alternative approaches described below.

### Dissociation of β: kinetics of subunit exchange

The rate constant of dissociation of a dimeric protein can be obtained directly from the kinetics of subunit exchange between two concentrated solutions of dimers provided that the exchange reaction results in a measurable change in an observable. To study subunit exchange in dimers of β, we labeled the protein with TMR at both sides of each interface (Figure 3A). Rhodamine dyes are known to form ground-state complexes characterized by a shift of the absorption spectrum to the blue and a low efficiency of fluorescence (29,30). Protein constructs labeled in this manner will be referred to as β-TMR_2_ to indicate that each monomer contains two TMR dyes. The crystal structure of β labeled in this manner with Alexa 488, a dye closely related to TMR, was obtained and shows that the overall structure of the dimer interface is the same as in the wild-type (wt) protein (21). The protein constructs used in the FCS experiments will be referred to as β-TMR_1_ to stress that each monomer contains only one TMR molecule. The fluorescence efficiency of β-TMR_2_ is reduced with respect to β-TMR_1_ due to the formation of TMR ground-state complexes around both clamp interfaces. This is evident in the fluorescence lifetimes measured with both constructs. Although the fluorescence decay of β-TMR_1_ can be fitted with two lifetimes (*τ*_1_ = 2.7 ns and *τ*_2_ = 1.1 ns), fitting the fluorescence decay of β-TMR_2_ requires an additional lifetime *τ*_3_ = 0.093 ns (Supplementary Figure S4) that we attribute to the ground-state rhodamine dimers that form around each interface. Short lifetimes of similar magnitude were observed for ground-state dimers of rhodamine covalently attached to a malarial protease substrate (31). We note that the intermediate lifetime (*τ*_2_) is present in both β-TMR_1_ and β-TMR_2_ and likely reflects an effect of the protein environment on the properties of the fluorophore.

To investigate subunit exchange between β dimers, we mixed 0.36 μM β-TMR_2_ and 1.8 μM wt-β in 50 mM NaCl Tris buffer and acquired time-resolved fluorescence decays at regular intervals over a total period of 40 h. Mixing β-TMR_2_ with excess unlabeled protein (wt-β) produces dimers that contain only one TMR molecule at each interface, and results in fluorescence recovery (Figure 3A). Indeed, we observed that the contribution of *τ*_3_ (quenched TMR) decreases, and the contribution of *τ*_1_ (unquenched TMR) increases, as the labeled and unlabeled clamps equilibrate (Figure 3B). The contribution of the intermediate lifetime (*τ*_2_) remained fairly constant during the exchange reaction, consistent with the observation that *τ*_2_ is present in both β-TMR_1_ and β-TMR_2_. We did not observe changes in the measured lifetimes or in the total number of acquired photons per trace in a control experiment with just 0.36 μM β-TMR_2_ (Supplementary Figure S4). This confirms that β does not spontaneously monomerize at these concentrations, and rules out photobleaching effects over the long experiments. In the mixing experiment, the pre-exponential factors are a direct measure of the number of molecules of TMR that contribute to each lifetime, and therefore, the relative amplitude that corresponds to *τ*_1_ (*α*_1_) is directly proportional to the concentration of mixed dimers (only one labeled subunit) and the relative amplitude of *τ*_3_ (*α*_3_) is directly proportional to the concentration of β-TMR_2_. The time-dependent changes of both *α*_1_ and *α*_3_ can be fitted with a monoexponential function with a relaxation time *τ*_x_ = 43 ± 3 h (Figure 3B). The relaxation time for the exchange reaction equals the reciprocal of the dimer dissociation rate constant (*τ*_x_ = 1/*k*_d_) because monomer association is orders of magnitude faster than dimer dissociation at protein concentrations much higher than the *K*_d_. In other words, the kinetics of subunit exchange is limited by the lifetime of the dimer (1/*k*_d_) because monomers associate into β dimers virtually instantaneously in the micromolar range. Based on these results, we conclude that the lifetime of the β dimer measured at room temperature and in a buffer containing 50 mM NaCl is 43 ± 3 h. This value is one order of magnitude higher than the lifetime of the PCNA trimer. The dissociation rate constant calculated from this lifetime is (6.5 ± 0.5) × 10^-6^ s^-1^. To obtain an estimate of the equilibrium dissociation constant, we recall that *K*_d_ = *k*_d_/*k*_a_, and that typical protein–protein association rate constants are in the 10^5^–10^6^ M^−1^ s^−1^ range (32,33). Based on these values, we estimate a *K*_d_ of 6.5–65 pM under the experimental conditions used in this study.

### Dissociation of β at the single-molecule level

Based on the results described above, we estimate that the dissociation constant of β is in the picomolar range. Spontaneous dissociation of β should therefore be observed upon dilution of the protein to concentrations in the low picomolar regime. Because FCS experiments with β-TMR_1_ are not feasible at these concentrations as discussed above, the doubly-labeled clamp (β-TMR_2_), which is essentially non-fluorescent in dimer form, was used to maximize the contrast between the dimer and the monomer (Figure 3A). Single-molecule fluorescence traces of 10 pM β-TMR_2_ measured right after dilution from a concentrated stock (*t* = 0) and after 8 h of incubation are shown in Figure 3C. The probability that a burst of fluorescence contains *k* detected photons/ms is plotted in the frequency histograms displayed in Figure 3C (middle). The ordinate is plotted in logarithmic scale to highlight bursts with large values of *k*, which are due to unquenched molecules of TMR. These events are rare because at 10 pM the observation volume contains only buffer molecules most of the time. A statistical analysis of the data acquired with pure buffer reveals that the probability of observing a burst of fluorescence >6 counts/ms is 4.1 ×10^−3^ (Figure 3C). Therefore, when we observe a burst with *k* > 6 photons/ms in the experiments with β-TMR_2_, the probability that the burst is due to the protein and not the background is >99.5%.

To quantitate the time-evolution of the histograms shown in Figure 3C (right), we plotted the fraction of bursts containing >6 photon counts/ms, *f* (*k* > 6), as a function of incubation time. To compare experiments performed on different days we normalized the values of *f* (*k* > 6) by the average count rate obtained during that particular experiment. The average of three independent experiments (Figure 3C) shows that the frequency of bursts with *k* > 6 increases over time with kinetics on the hour timescale. This reflects the increase in the concentration of protein monomers (fluorescent) as the dimer (non-fluorescent) dissociates. These data are not sufficient to calculate the dissociation constant of the dimer (*K*_d_) because we cannot relate the signal with the true concentration of monomers in the solution. Although the frequency of intense bursts is a measure of protein monomerization, the fraction plotted in Figure 3C is not necessarily a linear function of the concentration of monomer. In addition, we do not know if the single-molecule signal measured with 10 pM β-TMR_2_ at long times represents the signal of a completely dissociated sample, or whether a fraction of the protein remains stable as a dimer after equilibrium is reached. Despite these limitations, these data show that dissociation indeed occurs when β is diluted to 10 pM, and we are able to estimate a relaxation time for the equilibration process of the order of 3 h from a monoexponential fit to the data (Figure 3C, red line). This time is much shorter than the time measured in the mixing experiments (*τ*_x_ = 1/*k*_d_) because the relaxation time of a monomer/dimer system approaching equilibrium is related to the reciprocal of the sum of both the dissociation and association rates.

## DISCUSSION

### The formation of the PCNA trimer is a highly cooperative process

PCNA oligomerization experiments can be adequately described with a model that involves a trimer–monomer equilibrium characterized by a single equilibrium constant *K*_d_ = 2100 nM^2^. The stability of PCNA was previously investigated by Yao *et al.* by gel filtration chromatography (7). In this study, the authors investigated the stability of human PCNA (hPCNA) at 37°C and obtained a dissociation constant *K*_d_ = 21 nM by treating the raw data with equations commonly used in the analysis of receptor–ligand bimolecular interactions. A trimer–monomer equilibrium is not appropriately described by these models, and the thermodynamic equilibrium constant that describes the process should be expressed with units of concentration squared. For these reasons, we regard the value of *K*_d_ reported by Yao *et al.* as qualitative measure of the range of concentrations where the monomer co-exists in equilibrium with the trimer, and not as an appropriate descriptor of the dissociation equilibrium constant of PCNA. Although this prevents us from comparing the two *K*_d_ values directly, recall that the trimer and monomer exist at the same concentration when [M] = *K*_d_^1/2^ = 46 nM, and therefore, the raw data reported by Yao *et al.* is consistent with a dissociation constant of the same order of magnitude as the one we determined in this work.

Though a dimeric intermediate is not necessary to fit the data, the simultaneous encounter of three monomers is statistically improbable and therefore trimerization must occur by association of a third monomer to a sufficiently long-lived dimer. Our mathematical modeling shows that the dissociation constant of the transient dimer is at least one order of magnitude higher than the dissociation constant of the trimer. This indicates that association of the third monomer occurs with increased affinity, which is consistent with the fact that the last monomer forms two new interfaces closing the ring. Using mathematical modeling we estimated that the dissociation constant of the transient dimer must be >100 nM. Assuming a typical rate constant of 10^6^ M^−1^ s^−1^ for monomer–monomer association (32,33), we calculate that the lifetime of the dimer must be <10 s, i.e. at least three orders of magnitude lower than the lifetime of the trimeric ring (4.5 h). Whether this lifetime is long enough for a third monomer to associate with a dimer and close the ring before the dimer dissociates depends on protein concentration. Assuming again a typical association rate constant of 10^6^ M^−1^ s^−1^, we calculate an association rate of the order of 10^−3^ s^−1^ for protein concentrations in the nanomolar range. This suggests that, at these concentrations, successful encounters between the dimer and the third monomer will occur over timescales of ca. 10^3^ s, orders of magnitude larger than the estimated lifetime of the dimer. These arguments are consistent with our observations that trimer dissociation is nearly complete in equilibrated 1 nM PCNA solutions, and negligible in the micromolar range.

### The interface of β, but not PCNA, is stabilized by salt bridges

Experiments in buffers containing 1 M NaCl showed that salt concentration greatly affects the kinetics of dissociation of β, but has no effect on PCNA. High salt concentrations screen electrostatic interactions, and therefore, this indicates that electrostatic interactions between residues of opposite charge have a greater contribution to the stability of the interface of β than PCNA. The presence of potential intermolecular ion pairs was predicted when the crystal structure of the clamp was obtained and analyzed (15), and it has been reported that high salt concentrations promote subunit exchange in β (28). Our experiments at different NaCl concentrations are the first to address the role of salt bridges in a systematic and quantitative way. A quantitative treatment of electrostatic effects, however, is not straightforward because the β-clamp is not stabilized solely by electrostatic interactions. Stewart *et al.* showed that four nonpolar residues form a hydrophobic core that stabilize the interface of β, and mutation of two of these residues (I272A/L273A) weakens the interface such that β exists as monomers at low micromolar concentrations (34). Furthermore, a recent computational study reported calculated enthalpic contributions to the binding free energies and concluded that both β and PCNA are stabilized by van der Waals interactions, but that electrostatic interactions make an additional contribution to stabilizing the interface of β (35). Our experimental observations can be fully explained with this model, as salt bridges account for both the higher intrinsic stability of β in buffers of low ionic strength and the increased dissociation rate in buffers containing >150 mM NaCl.

### The three orders of magnitude difference in the dissociation constants of β and PCNA does not mirror their relative intrinsic stabilities

Is β intrinsically more stable than PCNA? For PCNA, the concentrations of trimer and monomer are identical when they equal *K*_d_^1/2^ = 46 nM. This value is three orders of magnitude higher than our upper estimate of the *K*_d_ of β (65 pM), indicating that indeed β is more stable than PCNA in solution. This is consistent with our observation that a 1 nM solution of β remains stable as a dimer even after 48 h of incubation, while PCNA dissociates almost completely into its constituting monomers. However, this large difference in equilibrium constants reflects greater differences in association rates of monomers than dissociation rates. Therefore, we argue that the thermodynamic dissociation constants do not represent true measures of the intrinsic stabilities of proteins with different oligomerization stoichiometry. Instead, the intrinsic stabilities of the clamps are better described by their intrinsic lifetimes (i.e. the inverses of the dissociation rate constants), which we calculated as 4.5 h for PCNA and 43 h for β. This indicates that despite the three orders of magnitude difference in the dissociation constants, the stability of β is just one order of magnitude higher than PCNA.

The dissociation of the trimer is cooperative, and therefore, the disassembly of PCNA in principle involves opening just two of the three interfaces. If the protein sequence and structure at the interfaces were identical for the two clamps, one could argue that a trimer should have a third of the stability of a dimer because there are three possible ways of opening two interfaces in the trimer. The fact that PCNA is one order of magnitude less stable than β suggests that this difference is not simply dictated by stoichiometry, but reflects differences in the strength of the interactions involved in holding the interfaces together. Recent force spectroscopy experiments carried out with clamps in which monomers were linked into a single polypeptide chain containing a single interface showed that the force required to open β is about three times larger than that required to open PCNA (35). This suggests that each of the interfaces of β is indeed more stable than each interface in PCNA. However, because the pulling experiments were performed out of equilibrium, and the measured forces were shown to depend on points of attachment and the direction of pulling, results cannot be directly translated into equilibrium binding energies in solution.

### Implications of clamp stability on biological function

As discussed above, we argue that intrinsic clamp lifetimes are the relevant parameters from the physiological point of view. Clamps do not assemble on DNA spontaneously, but must be loaded by enzymes called clamp loaders (2,4,36,37). For most clamps, this loading reaction includes stabilizing an open conformation of the clamp long enough to place the ring around DNA (37,38). Once loaded on DNA, a clamp, regardless of its subunit stoichiometry, must have a sufficient lifetime as a closed ring to allow the polymerase to synthesize the required segment of DNA and to coordinate Okazaki fragment maturation on the lagging strand. On completing synthesis, these stable clamps must be removed from DNA (39–42). Experiments with β and hPCNA at 37°C in a buffer containing 8 mM MgCl_2_ and 100 mM NaCl show that the lifetimes of the clamps on DNA in the absence of the clamp loaders are of the order of 1.7 and 0.5 h, respectively (7). These values are about one order of magnitude lower than the intrinsic lifetimes we measured for the isolated clamps in this study. These differences may merely reflect differences in the experimental conditions of the two studies (temperature, buffer composition and PCNA variant), or more importantly may indicate that rings are less stable once assembled on DNA. Given the dependence of the lifetime of the β-clamp on the ionic strength of the solution, the increase local charge when bound to DNA may destabilize this clamp. On the other hand, the lifetime of PCNA when free in solution was not influenced by ionic strength suggesting that more specific protein–DNA interactions may decrease the lifetime of the ring. The potential effect of the DNA on the stability of both β and PCNA will be the subject of future research.

Clamp dissociation requires breaking two monomer interfaces regardless of whether the clamp is a dimer or trimer, whereas loading clamps onto DNA requires opening a single interface (34,43,44). Breaking this single interface would be expected to destabilize a clamp relative to the closed ring form where all interfaces are intact and contribute to stability. Therefore, an additional function of the clamp loader may be to bind the individual monomers to prevent them from dissociating when the clamp is in an open conformation during the clamp loading process. This is supported by both biochemical and structural data for clamp loader–clamp complexes. PCNA subunit exchange rates are about the same for the free clamp and for the open clamp bound to RFC, suggesting that interactions with RFC maintain the stability of the oligomer when the clamp is in an open conformation (45). Structural data show that the five core clamp loader subunits contact one face of the clamp such that all of the clamp subunits interact directly with the clamp loader providing a mechanism by which the oligomeric state of the clamp is preserved on opening (46,47). An extreme example of clamp oligomer stabilization via clamp loader binding is PCNA from the archaeon, *Methanosarcina acetivorans*, that exists as monomers in solution. In this organism, the clamp loader binds the free monomers to catalyze the assembly of a trimeric ring on DNA (48). Thus, in all domains of life extensive interactions between the clamp loader and clamp stabilize the clamp oligomer in an open conformation to support clamp loading.

## SUPPLEMENTARY DATA

Supplementary Data are available at NAR online.

SUPPLEMENTARY DATA
