# Supplementary material for: Intrinsic stability and oligomerization dynamics of DNA processivity clamps
Source: Nucleic Acids Res. 2014 Apr 9;42(10):6476–86. doi: 10.1093/nar/gku255 (PMC4041429; doi:10.1093/nar/gku255)
Supplement: SUPPLEMENTARY DATA [file supp_42_10_6476__index.html]

Intrinsic stability and oligomerization dynamics of DNA processivity clamps — Intrinsic stability and oligomerization dynamics of DNA processivity clamps — Intrinsic stability and oligomerization dynamics of DNA processivity clamps — SUPPLEMENTARY DATA 

# Intrinsic stability and oligomerization dynamics of DNA processivity clamps

## SUPPLEMENTARY DATA

**Files in this Data Supplement:**

- Supplementary Data
