# Supplementary material for: Intrinsic stability and oligomerization dynamics of DNA processivity clamps
Source: Nucleic Acids Res. 2014 Apr 9;42(10):6476–86. doi: 10.1093/nar/gku255 (PMC4041429; doi:10.1093/nar/gku255)
Supplement: SUPPLEMENTARY DATA [file supp_gku255_nar-00356-h-2014-File005.pdf]

Intrinsic stability and oligomerization dynamics of DNA processivity clamps

Jennifer K. Binder, Lauren G. Douma, Suman Ranjit, David M. Kanno, Manas Chakraborty, Linda B. Bloom and Marcia Levitus

Supplementary Figure 1

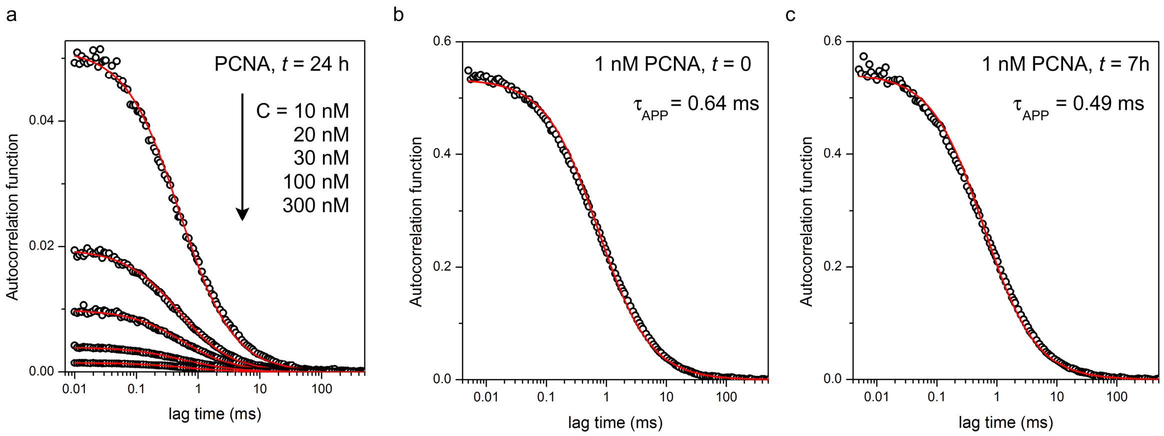

Examples of raw data and fitting curves. (a) FCS decays obtained with solutions of labeled PCNA that were prepared by diluting from a concentrated stock to the indicated final concentrations and equilibrating at room temperature for 24 h. The amplitudes of the decays are close to zero at concentrations above *ca*. 100 nM, and therefore experiments were performed by mixing labeled and unlabeled protein as indicated in the text. The apparent diffusion times (τ_{APP}) obtained by fitting these data with Eq. 1 (Materials and Methods) are τ_{APP} = 0.49 ms (C = 10 nM), 0.47 ms (C = 20 nM), 0.55 ms (C = 30 nM), 0.57 ms (C = 100 nM) and 0.60 ms (C = 300 nM). We note that these values are similar to the ones reported in Fig. 2B, which were obtained with solutions containing mixtures of labeled and unlabeled protein. (b-c) FCS decays obtained with a 1 nM solution of labeled PCNA right after diluting from a concentrated stock (t = 0) and after 7 h of incubation. The τ_{APP} values shown in the figures were obtained by fitting the experimental data with Eq. 1.

Supplementary Figure 2

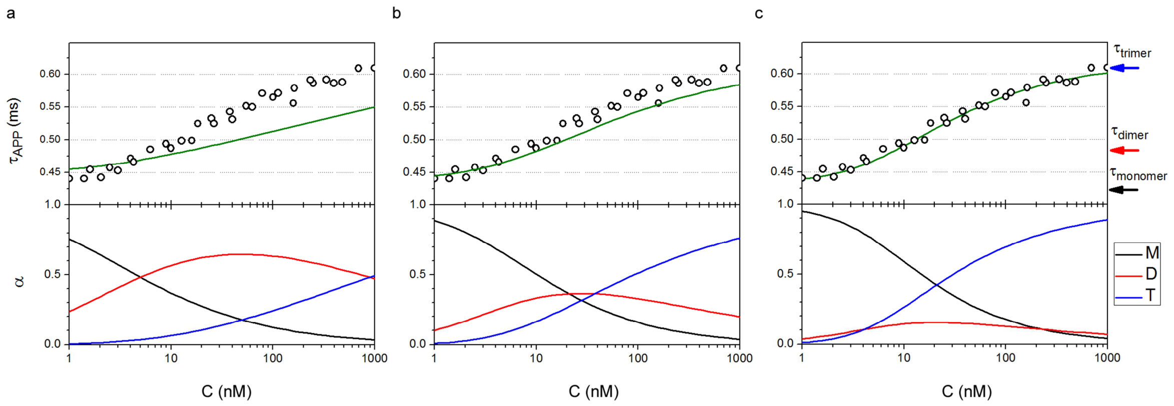

Analysis of the equilibrium data obtained with PCNA using a model that involves a dimeric intermediate. The black circles represent the τ_{APP} values recovered from the FCS decays of PCNA solutions containing 50 mM NaCl equilibrated for 24 hours (from Fig. 2B). The arrows represent the diffusion times of the pure trimer, dimer and monomer. The solid lines were obtained through mathematical modeling as described in Materials and Methods using different K_{d1} (T ⇔ D + M) and K_{d2} (D ⇔ 2M) values. In all cases the values of K_{d1} and K_{d2} were constrained by the apparent dissociation constant for the trimer-monomer equilibrium measured experimentally: $K_{d1}K_{d2} = K_d$ = 2,100 nM^2. Panel (a): K_{d1} = 145 nM and K_{d2} = 14.5 nM ($K_{d2} = K_{d1}$ /10), panel (b): K_{d1} = 45.8 nM and K_{d2} = 45.8 nM ($K_{d2} = K_{d1}$), and panel (a): K_{d1} = 14.5 nM and K_{d2} = 145 nM ($K_{d2} = 10K_{d1}$). The fractional concentration profiles at the bottom of each graph were calculated as $\alpha_1 = \frac{[M]}{3C}$ for the monomer, $\alpha_2 = \frac{2[D]}{3C}$ for the dimer, and $\alpha_3 = \frac{[T]}{C}$ for the trimer. Modeling shows that only values of $K_{d2} \geq 10\ K_{d1}$ fit the experimental data, and K_{d1} = 14.5 nM and K_{d2} = 145 nM give the highest concentration of dimer that is compatible with the experimental points.

Intrinsic stability and oligomerization dynamics of DNA processivity clamps

Jennifer K. Binder, Lauren G. Douma, Suman Ranjit, David M. Kanno, Manas Chakraborty, Linda B. Bloom and Marcia Levitus

Supplementary Figure 3

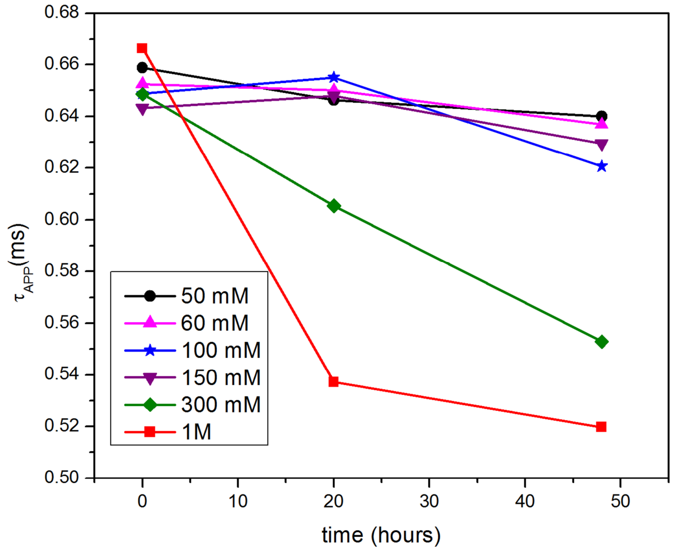

Apparent diffusion times (τ_{APP}) obtained from the FCS decays measured with 1nM β in Tris buffer containing the indicated concentrations of NaCl. The time axis indicates the incubation time.

Intrinsic stability and oligomerization dynamics of DNA processivity clamps

Jennifer K. Binder, Lauren G. Douma, Suman Ranjit, David M. Kanno, Manas Chakraborty, Linda B. Bloom and Marcia Levitus

Supplementary Figure 4

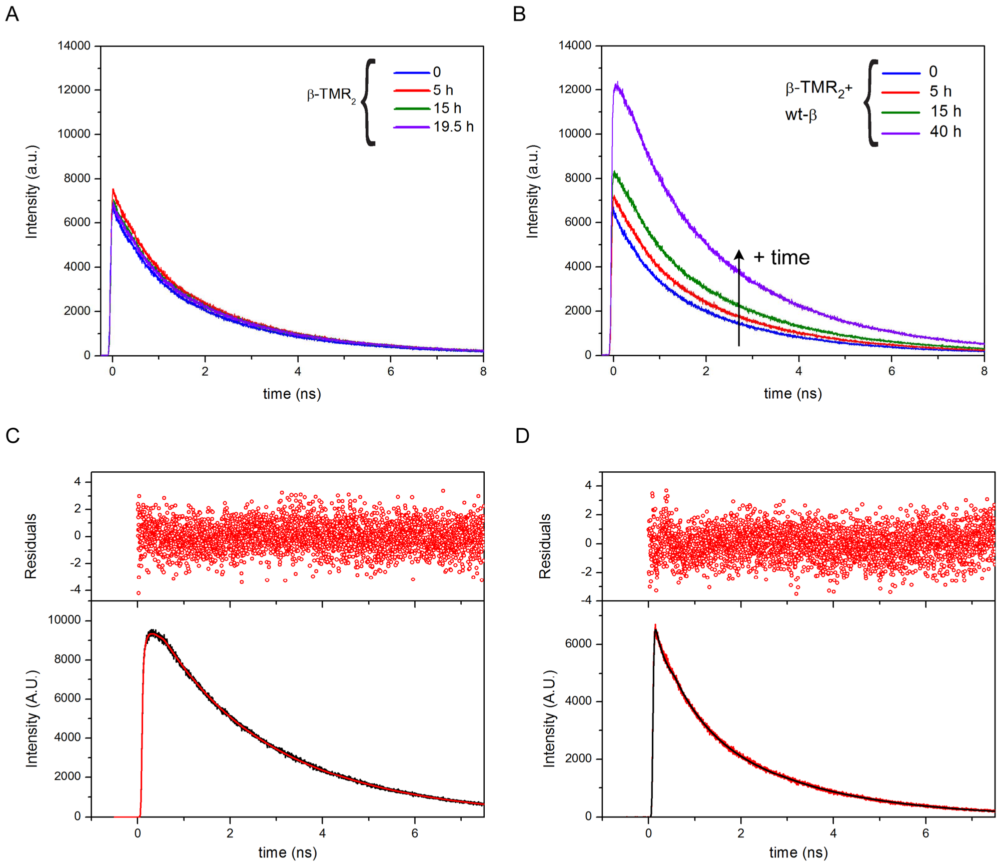

Time-resolved fluorescence decays of 0.36 μM β-TMR_2 collected at different incubation times with (B) or without (A) 1.8 μM wt-β. No changes in intensity or lifetime were observed with β-TMR_2 alone (A), ruling out dimer dissociation and potential photobleaching artifacts. With wt-β present (B), we observe an increase in the total number of collected photons over 40 h of incubation time due to subunit exchange. The normalized decays that correspond to these data are shown in Fig. 3B in the manuscript. The decay of β-TMR_1 (C) can be fitted with two lifetimes (τ_1 = 2.7 ns and τ_2 = 1.1 ns), while the decays of β-TMR_2 (D) require a third exponential term with τ_3 = 0.093 ns.
